# *TrpA1* Regulates Thermal Nociception in *Drosophila*

**DOI:** 10.1371/journal.pone.0024343

**Published:** 2011-08-31

**Authors:** G. Gregory Neely, Alex C. Keene, Peter Duchek, Elaine C. Chang, Qiao-Ping Wang, Yagiz Alp Aksoy, Mark Rosenzweig, Michael Costigan, Clifford J. Woolf, Paul A. Garrity, Josef M. Penninger

**Affiliations:** 1 Neuroscience Program, Garvan Institute of Medical Research, Darlinghurst, Sydney, New South Wales, Australia; 2 Institute of Molecular Biotechnology of the Austrian Academy of Sciences, Vienna, Austria; 3 Biology Department, New York University, New York City, New York, United States of America; 4 National Center for Behavioral Genomics, Department of Biology, Brandeis University, Waltham, Massachusetts, United States of America; 5 Program in Neurobiology, Children's Hospital Boston and Department of Neurobiology, Harvard Medical School, Boston, Massachusetts, United States of America; University of Missouri, United States of America

## Abstract

Pain is a significant medical concern and represents a major unmet clinical need. The ability to perceive and react to tissue-damaging stimuli is essential in order to maintain bodily integrity in the face of environmental danger. To prevent damage the systems that detect noxious stimuli are therefore under strict evolutionary pressure. We developed a high-throughput behavioral method to identify genes contributing to thermal nociception in the fruit fly and have reported a large-scale screen that identified the Ca^2+^ channel *straightjacket* (*stj*) as a conserved regulator of thermal nociception. Here we present the minimal anatomical and neuronal requirements for *Drosophila* to avoid noxious heat in our novel behavioral paradigm. Bioinformatics analysis of our whole genome data set revealed 23 genes implicated in Ca^2+^ signaling that are required for noxious heat avoidance. One of these genes, the conserved thermoreceptor *TrpA1*, was confirmed as a bona fide “pain” gene in both adult and larval fly nociception paradigms. The nociceptive function of *TrpA1* required expression within the *Drosophila* nervous system, specifically within nociceptive multi-dendritic (MD) sensory neurons. Therefore, our analysis identifies the channel TRPA1 as a conserved regulator of nociception.

## Introduction

Acute and chronic pain will affect most people at some stage in their lives [1]. Chronic pain in particular represents an unmet clinical need [2]. Nociception, the neuronal sensory and processing apparatus that relays the perception of acute pain, allows an organism to avoid potential tissue damage and death, and many genes regulating this process are conserved across phyla [3]. Transient Receptor Potential (TRP) channels are a family of sensory ion channels that were first identified in *Drosophila melanogaster* [4], [5], and have subsequently been identified as critical mediators of nociception in mammals [6]. The TRP family channel *painless* was identified in *Drosophila* using a larval heat probe assay [7]. While *painless* has no mammalian orthologue [8], [9], it is possible that other components of the *Drosophila* nociception apparatus are indeed conserved from flies to mammals.

To interrogate *Drosophila* for conserved genes that regulate nociception, we developed a high-throughput screening procedure [3]. This behavioral system utilizes the robust ability of adult fruit flies to rapidly avoid noxious heat. This system has led to the identification of hundreds of candidate fly “pain” genes, for example *straightjacket* (*stj*), as regulators of nociception behavior in *Drosophila* [3]. Here we show this innate avoidance behavior is independent of other sensory modalities known to promote avoidance responses, such as vision, olfaction, CO_2_ perception, hearing, and taste and requires intact antennae and proboscis for a full response. Importantly, *painless*-expressing neurons, but not the mushroom body which is required for sub noxious thermo-preference [10], are a necessity for this behavior. We further provide genetic evidence that one of the candidate pain genes, *TrpA1*, is a bona fide mediator of thermal nociception in the fly. Tissue-specific RNAi knockdown of *TrpA1* revealed that TrpA1 functions in nociceptive multi-dendritic (MD) sensory neurons. Thus, TRPA1 regulates the *Drosophila* behavioral response to a noxious thermal insult. Combined with TRPA1's role in chemical nociception, our results identify TRPA1 as an evolutionary conserved regulator of polymodal nociception.

## Results

### Set-up of a high-throughput system to screen for nociception in *Drosophila*

We recently developed a high-throughput assay to perform an *in vivo* genome-wide pain screen in *Drosophila* [3]. Here we report the detailed set-up and anatomical/neuronal requirements for this novel behavioral paradigm, data we believe are essential for the field and future use of this system. In preliminary pilot studies to address nociceptive responses, we found the *Drosophila* response to noxious heat exposure more reliable and robust compared to mechanical pain paradigms (not shown). Furthermore, while the commonly used *Drosophila* larval nociception paradigm has proven suitable for identifying genes required for nociceptive behavior [7], it is labor intensive and requires evaluations of larval responses not compatible with large-scale applications.

**Figure 1:**
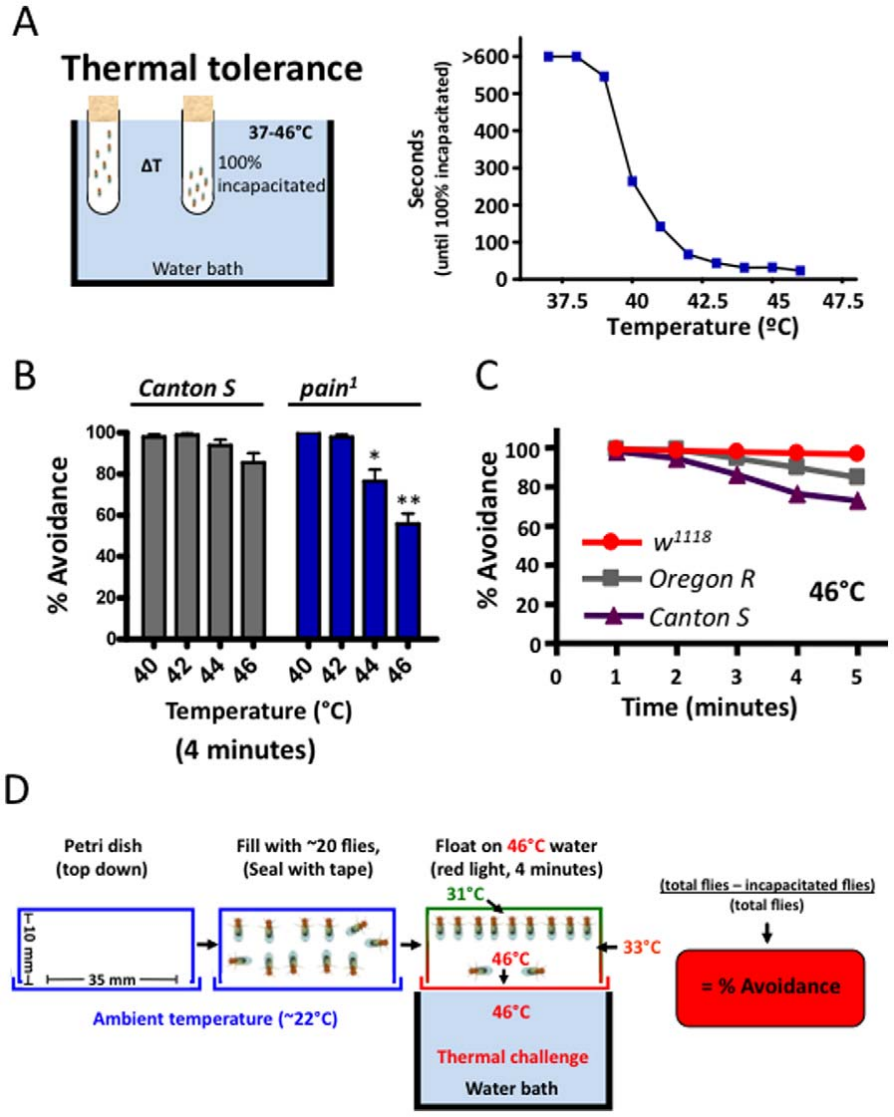
Development of an avoidance assay to noxious heat in adult *Drosophila*. (**A**) Temperature-response profiles to identify acutely noxious temperatures for adult *Drosophila*. Experimental setup is depicted (left panel) and mean dose-response values are presented (right panel). (**B**) *painless* mutants (*pain^1^*) are impaired at avoiding noxious temperatures above 42°C compared to wild-type *Canton S* controls . wild type (*Canton S*) and pain^1^ avoidance responses are presented. Data are presented as mean +/− SEM. * P<0.05, ** P<0.01 by Student's t test. Data are presented as mean +/− SEM. (**C**) Time course of high temperature (46°C) thermal avoidance responses for 3 common *Drosophila* laboratory strains reveals robust avoidance responses in all strains tested. In all indicated experiments, n>20 progeny per group. (**D**) Schematic for high-throughput heat nociception using adult *Drosophila*. The final setup used for assaying heat nociception in *Drosophila* is depicted. Flies are placed into the experimental chamber and the chamber sealed with scotch tape. Flies are rested for at least 30 minutes in the dark, and the chambers then floated on a 46°C water bath for 4 minutes. Immobilized flies are counted as “incapacitated”. Moreover, total fly numbers are recorded to calculate the values for percent avoidance.

To develop a high-throughput screening system in adult *Drosophila*, we first defined acute noxious heat thresholds. By subjecting flies to increasing gradients of noxious heat exposure, we found that flies rapidly became incapacitated following exposure to temperatures above 40°C (Figure 1A) and continued exposure to such temperature (>2 minutes) was lethal (not shown). Temperatures below 39°C did not incapacitate flies within the 10 minute time course. This thermal tolerance profile was very similar to that reported by others, indicating a common thermal tolerance threshold across *Drosophila* strains and experimental paradigms [11]. Since nociception is the sense an animal employs to detect and avoid potential harm, and because exposure to temperatures about 40°C were acutely harmful to *Drosophila*, we exploited this rapid incapacitation as a means of large-scale screening for nociception behavior. We therefore developed an experimental test chamber where the temperature of the base surface of the chamber could be controlled and rapidly increased when required, giving flies a choice between a hot and potentially lethal surface and a surface that remained close to room temperature.

Using this paradigm, we found that wild-type *Canton-S* flies rapidly avoid all noxious temperatures tested. Flies mutant for the classical *painless* (*pain^1^*) gene could avoid surfaces heated up to 42°C, but failed avoiding the surface if the temperature is ≥42°C (Figure 1B). These differences between wild-type and *pain^1^* flies were greatest at 46°C. To assess a potential influence of the genetic background, we assayed three different laboratory *Drosophila melanogaster* strains. *Canton-S*, *Canton-S^w1118^*, and *Oregon-R* strains all rapidly and reproducibly avoided the heated surface during the course of the experiment (Figure 1C). Our final experimental apparatus involves an inverted petri dish with ∼20 flies, sealed with tape, and floated on a 46°C water bath (Figure 1D). The chamber is 35 mm wide with a 10 mm distance between the hot and warm surfaces. The bottom heated surface reaches 46°C within 15 seconds of the experiment, while the internal top and middle surfaces reach 31°C and 33°C by the end of the 4 minute test period. The maximum internal air temperature recorded during the experiment was 31°C. Using this system we can generate a % avoidance value for each genotype tested (Figure 1D). Thus, adult *Drosophila* exhibit a robust and highly reproducible innate avoidance response to noxious heat, and in the fly this response is dependent on the *painless* gene.

### Mapping anatomical structures critical for high temperature nociception in *Drosophila*

**Figure 2:**
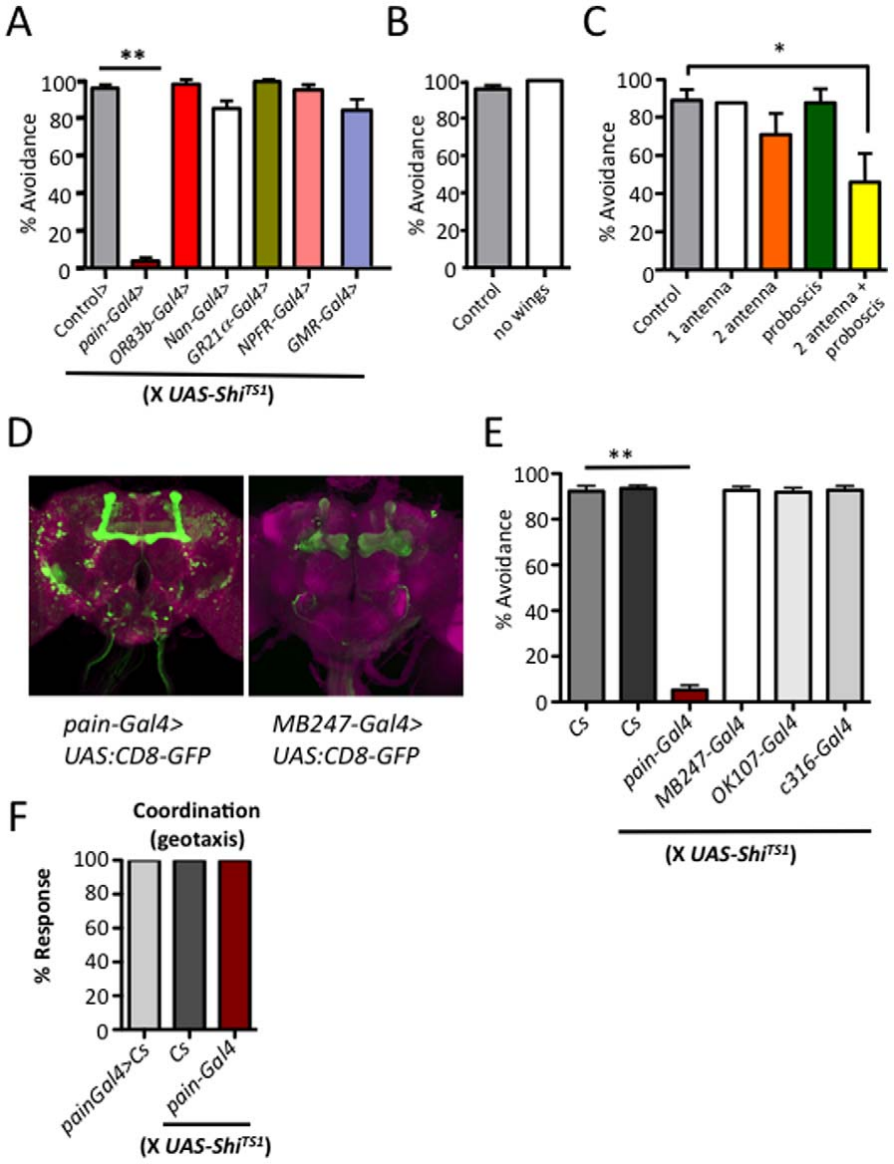
The *Drosophila* antenna, proboscis, and *painless* expressing neurons are required for avoidance of noxious heat. (**A**) Synaptic output from *painless* expressing neurons (*painless*-*Gal4*>UAS-*Shi* ^ts1^), but not other sensory modalities tested, is required to avoid noxious heat. Sensory nerves mediating olfaction (*OR83b-Gal4>UAS-Shi^ts1^*), hearing and hygrosensation (*Nan-Gal4>UAS-Shi^ts1^*), the CO_2_ sensing apparatus (*GR21α-Gal4>UAS-Shi^ts1^*), bitter avoidance (*NPFR-Gal4>UAS-Shi^ts1^*), and vision (*GMR-Gal4>UAS-Shi^ts1^*) are not required for thermal nociception. Control line is *w^1118^*×UAS-*Shi* ^ts1^. (**B**) Wings are not required for noxious thermal avoidance in *Drosophila*. Wings were dissected off and flies were tested for the ability to avoid noxious temperature. (**C**) Antenna and proboscis cooperate to promote avoidance of noxious temperature. One or both antenna and the proboscis were dissected from wild type flies and then tested for avoidance of noxious temperature. (**D**) *pain-Gal4* driving *UAS-CD8:GFP* labels *painless* expressing neurons in the mushroom body and other regions of the adult fly brain. *MB247-Gal4>UAS-CD8:GFP* labels the mushroom body. (**E**) Synaptic output from *painless* expressing nerves (*pain-Gal4>UAS-Shi^ts1^*), but not neurons from the mushroom body (*MB247-Gal4>UAS-Shi^ts1^, OK107-Gal4>UAS-Shi^ts1^*) or mushroom body associated DPM neurons *(c316-Gal4>UAS-Shi^ts1^)*, is required for thermal nociception in adult *Drosophila*. *Cs* (*Canton S*). (**F**) Synaptic silencing of painless expressing nerves (*pain-Gal4>UAS-Shi^ts1^*) does not affect basic motor coordination as assayed by a negative geotactic assay. All experiments involving *UAS-shibire^TS1^* were pre-incubated at 30°C for 1 hour to induce *shibire*-mediated synaptic silencing. Data are presented as mean values +/− SEM. ∼20 flies tested per group, in replicates of at least four cohorts. In all experiments adult flies were challenged with 46°C as outlined in Figure 1D. * P<0.05, ** P<0.01 (Student's t-test).

Little is known about the anatomy regulating the behavioral response to an acute noxious insult in adult *Drosophila* [12]. We therefore assayed whether noxious heat avoidance requires sensory and higher order neurons implicated in other avoidance behaviors. Blocking synaptic transmission in neurons by driving *UAS-Shi^ts1^* (*Shibire* ^ts1^; a temperature sensitive dynamin mutant [13]) in subsets of neurons controlling olfaction (*OR83b-Gal4*), hearing and hygrosensation (*nan-Gal4*), and vision (*GMR-GAL4*) did not affect nociceptive behaviors, indicating that avoidance of noxious heat is independent of these sensory modalities (Figure 2A). Furthermore, thermal nociception did not require neurons previously implicated in other aversive behaviors such as the *Gr21a*-expressing CO2-sensitive olfactory neurons [14], or neurons expressing neuropeptide F (*NPF-Gal4>UAS-Shi^ts1^*) or its receptor (*NPFR-Gal4>UAS-Shi^ts1^*) which have been implicated in bitter taste avoidance [15]. Importantly, thermal nociception was dependent on synaptic transmission in *painless*-expressing neurons (Figure 2A). Taken together, these findings indicate that thermal nociception in adult *Drosophila* requires painless expressing neurons but does not rely on sensors or cells previously implicated in vision, olfaction, hearing, CO2 sensing, hygrosensation, or bitter avoidance.

To map the anatomy of thermal nociception in adult fly, we surgically removed the *Drosophila* wings, proboscis and antennae to determine if these structures are involved in the response to avoid noxious heat. We found flies lacking wings respond normally to noxious heat, indicating that wings are not required for thermal nociception (Figure 2B). Previous studies have implicated the third-antennal segment as a component of the sensory apparatus required for rapid avoidance of elevated temperatures between 25°C and ∼33°C [16], [17]. We observed a modest requirement for antenna in avoidance of noxious heat, which appeared to cooperate with the proboscis in the observed avoidance response (Figure 2C). Thus, the antenna and proboscis are candidate organs for sensing noxious heat.

In the adult fly, it has been reported that *painless* is expressed in the wing, proboscis, leg, and in central brain neurons that include the mushroom bodies ([18] and Figure 2D). Mushroom bodies have been implicated in thermal preference in the more long-term (15 minutes) behavioral response to sub-noxious temperatures [10]. To silence mushroom body neurons, we used the mushroom body drivers *OK107-Gal4* [19], [20] and *MB247-Gal4*, two of the same *Gal4* driver implicated in sub-noxious thermal preference [10], both of which express broadly throughout the mushroom bodies [21]. In addition we used the mushroom body associated DPM driver *c316-Gal4* [22]. Using the *Gal4/UAS* system to express *Shi* ^ts1^, we again found that synaptic silencing of *pain-Gal4* neurons was sufficient to abolish the heat avoidance response. However, silencing of the mushroom body itself, or mushroom body associated neurons, had no effect on the avoidance response to noxious heat (Figure 2E). Flies with *UAS-Shi^ts1^*, or *pain-Gal4* alone showed wild-type avoidance, indicating the avoidance defects observed in *pain-Gal4;UAS-shi^ts1^* are due to the silencing of *pain*-expressing neurons. Importantly, silencing of *painless*-expressing neurons did not result in general coordination defects, as assessed by negative geotactic response (Figure 2F). Thus, *pain-Gal4* expressing cells outside of the mushroom bodies are required for adult *Drosophila* to sense noxious heat.

### Genes implicated in calcium signaling are over-represented among candidate heat nociception genes

**Figure 3:**
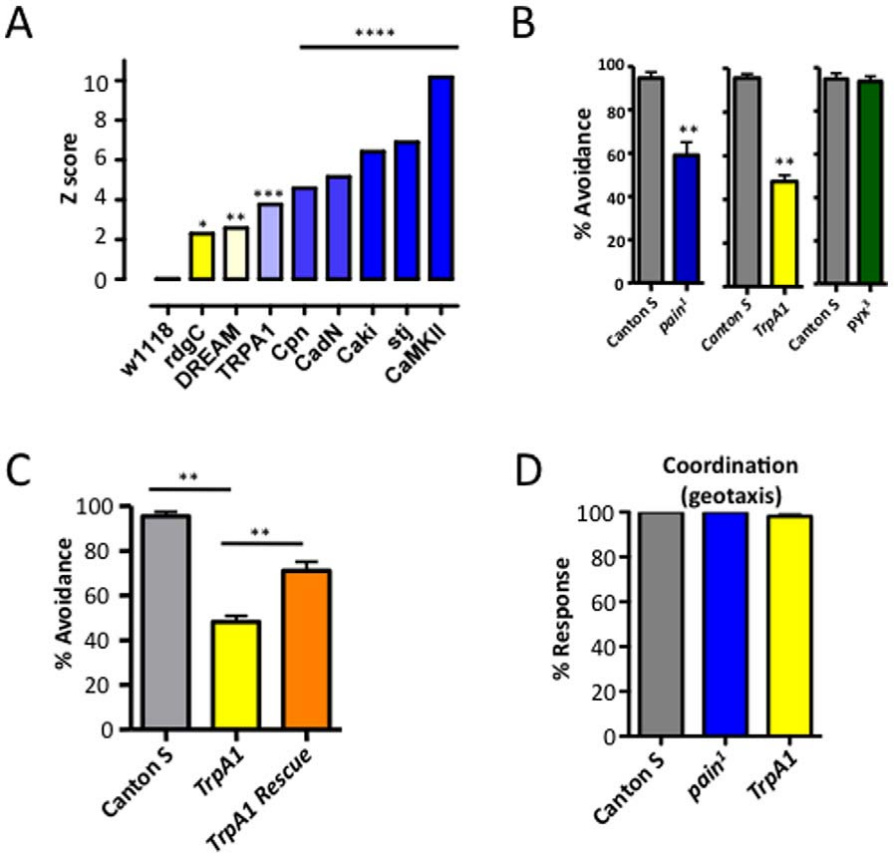
TrpA1 is a novel *Drosophila* nociception gene. (**A**) Thermal avoidance of a select list of *elav-Gal4*×*UAS-IR* lines targeting genes involved in Ca^2+^ signaling are depicted with Z-score and calculated significance. (**B**) *TrpA1* and *painless (pain^1^)*, but not *pyrexia* (pyx^3^) mutant flies exhibit defects in thermal nociception in adult *Drosophila*. (**C**) Re-introduction of *TrpA1* on the *TrpA1* mutant background is sufficient to rescue the defective adult thermal nociception response, establishing that the observed thermal nociception defect is specific to *TrpA1* expression and not the result of secondary effects. (**D**) Loss of *painless* or *TRPA1* does not affect basic motor coordination as assayed by a negative geotactic assay. Data are presented as mean values +/− SEM. ∼20 flies tested per group, in replicates of at least four cohorts. In all experiments ∼20 flies were tested per group in replicates of at least four. In all experiments adult flies were challenged with 46°C as outlined in Figure 1D. * P<0.05, ** P<0.01, *** P<0.001, **** P<0.0005 (based on (**A**) Z score and (**B–C**) Students t-test).

We have previously reported 580 candidate genes involved in nociception in *Drosophila* [3]. Based on these primary hits, we performed hypergeometric enrichment analysis using KEGG pathways and Broad Institute C2 gene sets to identify groups of genes that were over-represented in our genome wide screen for nociception. One prominent gene category identified by this analysis was calcium signaling (Figure 3A, Table S1 for full data set). For instance, Calphotin (Cpn; CG4795) is a calcium binding molecule implicated in rhabdomere development. Moreover, we hit the calcium channel subunit *straightjacket* as we have reported in detail elsewhere [3]. We also found the fly ortholog of DREAM (Calsenilin, KChip3), a calcium-regulated central pain gene in mice [23], the calcium regulated cell adhesion molecule CG7100 (CadN), a calcium-dependent EF hand protein serine/threonine phosphatase (CG6571, rdgC), CAMKII, a gene that has also been linked to the modulation of TRPV1 channel function [24], as well as the CAMKII activator Caki (CG6703, Camguk), a member of the MAGUK family of proteins that contains a CaMKII-like domain and participates in regulation of calcium channel function in other species [25]. Finally, our approach identified *dTrpA1* (*TrpA1*), the *Drosophila* ortholog of the chemical and cold sensing human TRPA1, a Ca^2+^-permeable non-selective cation channel implicated in acute chemical pain and cold hypersensitivity in rodents [26], [27] and infrared sensation in snakes [28].

### TRPA1 is required for thermal nociception in both larvae and adult *Drosophila*

*TrpA1* is a member of the *TRPA* subfamily of TRP channels. *Drosophila* TRPA1 is the fly ortholog of human TRPA1 and the two proteins share ∼33% overall sequence identity, whereas other insect *TRPA* family members such as *painless* and *pyrexia* belong to a distinct subclass of TRPAs lost during vertebrate evolution [8]. *Drosophila* TRPA1 has been found to act as a warmth-activated ion channel required for thermotaxis at non-noxious temperatures and to act as a receptor mediating avoidance of reactive electrophilic chemicals [8], [29], [30]. To definitively demonstrate that fly *TrpA1* is essential for noxious heat avoidance, we tested whether classical mutants for *TrpA1* exhibit impaired avoidance of noxious heat. Consistent with the RNAi knockdown results, animals homozygous for a loss-of-function mutation in *TrpA1* (*TrpA1^ins^*) [30] failed to avoid noxious temperature to a level similar to *painless* mutants (Figure 3B). To determine if the observed *TrpA1* adult pain phenotype was the result of increased temperature-induced paralysis at 46°C, we exposed *TrpA1* and control flies to a chamber set to 46°C and recorded the kinetics of temperature-induced paralysis. In support of an adult pain phenotype, we observed no difference in temperature-induced paralysis between these lines (36.6+/−1.4 seconds control, 33.2+/−1.3 seconds *TrpA1* flies, n = 12, not significant by t test). The avoidance defect was rescued by reintroduction of a *TrpA1* minigene into the *TrpA1* mutant background, confirming that the observed defect in thermal nociception was due to the disruption of *TrpA1* (Figure 3C). In contrast, we did not observe a noxious heat avoidance phenotype in a mutant for another *Drosophila* TRPA, *pyrexia* (*pyx^3^*) (Figure 3B), previously implicated in high temperature (40°C) thermal tolerance [31]. Importantly, painless and TRPA1 mutant flies exhibit normal coordination as assessed by a negative geotactic response (Figure 3D). Thus, both *painless* and *TrpA1* are required for avoidance of noxious heat in adult *Drosophila*.

**Figure 4:**
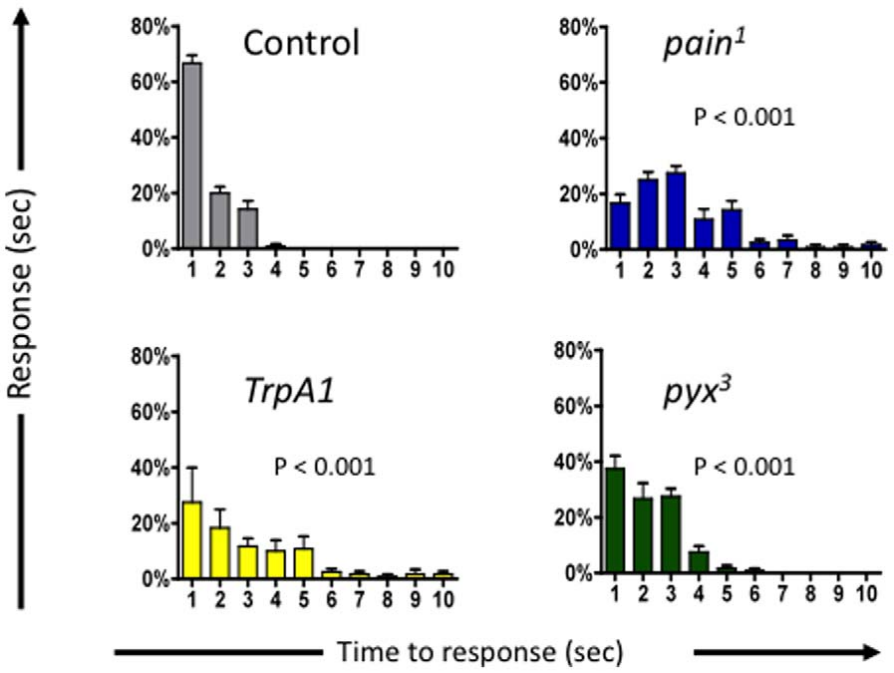
Both *Painless* and *TrpA1* are required for thermal nociception in the *Drosophila* larvae. *TrpA1 (TrpA1^ins^)*, *painless (pain^1^)*, and *pyrexia* (*pyx^3^*), mutant larvae were tested for their response to high temperature using a 46°C probe. % Response for each genotype is presented at each second within a ten second test period. A Kruskal-wallis non-parametric test for median comparison followed by the Dunn's post-hoc test was used for statistical analysis. P values are indicted in the panels. Data are presented as mean values +/− SEM. In all experiments ∼20 larvae were tested per group in replicates of at least four.

As *TrpA1* participates in thermal nociception in adult *Drosophila*, we also assayed whether *TrpA1* is involved in thermal pain behavior in larvae (Figure 4). Larvae were gently touched with a 46°C probe and avoidance time was measured. While control larvae showed a rapid response to noxious heat, the *TrpA1* mutants showed a significantly diminished capacity for thermal nociception (Figure 4). In contrast to adult flies, *pyx^3^* mutant larvae also showed an impairment in this larval nociception assay. Thus, we report a key role for *Drosophila TrpA1* in larval and adult thermal nociception, i.e. *TrpA1* is a bona fide pain gene in *Drosophila*.

**Figure 5:**
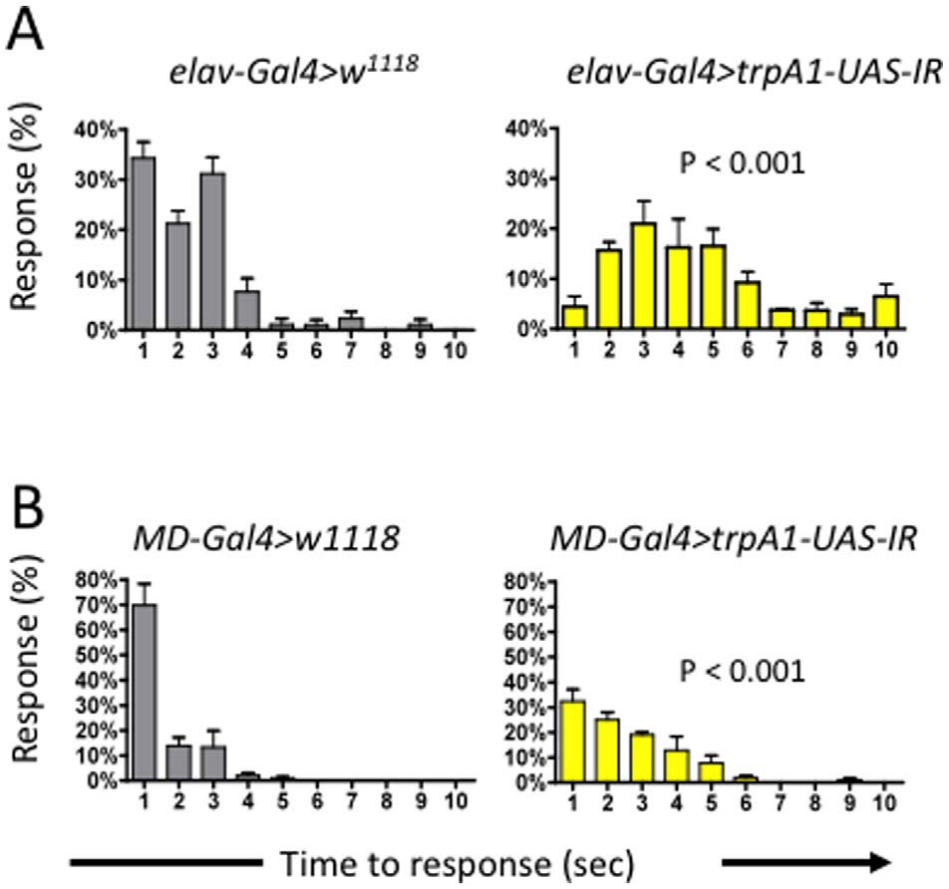
TrpA1 functions in multi-dendritic sensory neurons in the larval pain response. Larvae pain response profiles in (**A**) *elav-Gal4>TrpA1-UAS-IR* lines to target *TrpA1* in all neurons and (**B**) *MD-Gal4>TrpA1-UAS-IR* to target *TrpA1* in sensory neurons. % Response to a 46°C heat probe is presented for each genotype at each second within a ten second test. A Kruskal-wallis non-parametric test for median comparison followed by the Dunn's post-hoc test was used for statistical analysis. P values are indicted in the panels. Data are presented as mean values +/− SEM. In all experiments ∼20 flies were tested per group.

Blocking neurotransmission from multi-dendritic (MD) sensory neurons reduces thermal nociception responses in *Drosophila* larvae [7]. To localize the site of *TrpA1* function in thermal nociception we therefore employed *UAS-TrpA1-IR* flies. Driving *TrpA1*-RNAi in all neurons using *elav-Gal4* (*elav-Gal4>TrpA1-UAS-IR*) resulted in significant impairment of the larval thermal nociception response (Figure 5A). Driving *TrpA1* RNAi in MD-sensory neurons alone (*MD-Gal4>TrpA1-UAS-IR*) also impaired the larval thermal nociception response (Figure 5B). These results indicate that TRPA1 is, at least in part, acting in multi-dendritic sensory neurons.

## Discussion

Our novel high-throughput system for assessing nociception behavior in adult *Drosophila* has allowed us to screen the entire *Drosophila* genome to examine the neural basis of thermal nociceptive behavior. Here we describe neuronal requirements and anatomical structures involved in this innate behavioral paradigm of thermal nociception. We also provide first experimental proof that *TrpA1* functions in sensory neurons as a novel component of the *Drosophila* thermal nociception apparatus.

TRPA1 is recognized as a key component of the nociception apparatus in mammals. Not only do mice mutant for TRPA1 show defects in nociception, a mutation in TRPA1 underlies a human episodic pain syndrome [32]. At the molecular level, the role of the mammalian TRPA1 as a receptor for electrophilic chemicals and other irritants is well established [27], and the mechanism of how TRPA1 functions in chemical nociception is highly conserved between humans and flies [8]. Mammalian TRPA1 is also implicated in cold nociception, at least under pathological conditions [26], suggesting it contributes to multiple nociceptive sensory modalities. In *Drosophila*, TrpA1 has been shown to act as an internal thermosensor regulating temperature preference at non-noxious temperatures [30] and as a chemoreceptor for noxious electrophilic irritants [8]. That TRPA1 participates in both subnoxious thermal preference [30] and avoidance of noxious heat is an interesting observation. This could reflect distinctions between the mechanisms that control responses to steep versus shallow gradients, with thermal preference behavior involving long-term (>15 minute) exposure to shallow (∼0.5°C/mm) temperature gradients [30], while the current assay assesses behavioral short-term (<4 minutes) responses to much steeper gradients (∼4.5°C/mm by assay endpoint). These potential distinctions are currently being explored in our laboratories.

Our study expands the role of *Drosophila* TrpA1 signaling to responses to noxious heat. Combined with recent work demonstrating its role in chemical nociception [8], TrpA1 mediates both chemical and thermal nociception in *Drosophila*. Thus, while the temperature-responsiveness of TRPA1 has undergone significant diversification within the animal lineage, from being heat-activated in flies [33] and snakes [28] to potentially cold-responsiveness in mammals [26], [34], a role for TRPA1 in polymodal nociception has been retained from flies to mammals.

In addition to *TrpA1*, *painless* and *stj* (and to a lesser extend pyrexia in larvae) are required for the pain response in *Drosophila*. Thus it appears that, similar to mammals, multiple cation channels are involved in pain-responses in flies. While *painless* and *pyrexia* encode insect-specific TRPA-channels, *TrpA1* and *stj* are conserved in humans. Both TRPA1 and *stj* (*CACNA2D3* in humans) have been implicated in human nociception suggesting core genetic regulators of nociception are strongly conserved from flies to humans [3].

The ability to perform high-throughput screening for mediators of nociception has until now been limited to *in vitro* models and not intact behaving animals. Our *in vivo* system expands the toolbox available for pain researchers. Coordinated use of this fly system could accelerate the identification of new compounds to be short listed for validation in mammalian models of pain. Using this novel paradigm, we have now described hundreds of new candidate “pain” genes, opening the field up to multiple new candidate analgesic targets. Among these genes, our RNAi-screen identified multiple genes that are predicted to play a role in Ca^2+^ signaling such as TRPA1. Future analysis of these genes should provide valuable insight into the neural basis of nociceptive behavior and the role of calcium signaling in pain.

## Materials and Methods

### Fly stocks

*UAS-IR* transgenic fly lines and Canton S, Oregon R, and w^1118^ flies were obtained from the VDRC RNAi library [35]. *elav* with UAS-Dicer 2 was a gift from B. Dickson (Institute for Molecular Pathology) [35]. *painless (EP(2)2451)*, *pain-Gal4* and *MD-Gal4* were gifts from D. Tracey (Duke Medical School). *Pyrexia^3^* was a gift from J. Kim (Korea Advanced Institute of Science & Technology). *Nan-Gal4* was a gift from C. Kim (Chonnam National University). *GR21a-Gal4* was a gift from D. Anderson (Cal Tech). *NPFR-Gal4* was obtained from P. Shen (University of Georgia). *OR83b-Gal4* was a gift from L. Vosshall (Rockefeller University). *MB247-Gal4* was generated by Robert Schulz [21], OK107*-Gal4* was described in [19] and characterized further in [20], and c316*-Gal4* was described in ref. [22]; all three lines were provided by Scott Waddell (University of Massachusetts Medical School). *Eyeless-Gal4*, *gmr-Gal4*, *UAS-Shibire^ts1^*, and *UAS-CD8-GFP* were obtained from Bloomington. *dTrpA1^ins^* and *TrpA1* rescue lines have been previously described [30].

### Behavior experiments

For adult avoidance of noxious heat, ∼20 four day old flies were placed into a behavioral chamber (35 mm×10 mm Petri dish; Nunclon) and the chamber was sealed with scotch tape. Flies were rested for at least 30 minutes in the dark. The chambers were then floated on a 46°C water bath for 4 minutes. The bottom of the chamber was heated to 46°C over 15 seconds by floating on a water bath while the sub-noxious zone was measured to be 31°C (inner top of chamber) and 33°C (middle edge of the chamber) at the end of the 4 minute experiment. Chamber temperature was monitored using an electronic thermometer (Testo 925, Germany) coated in heat sink gel (RS components, UK). Chambers were then removed from the water and immobilized flies were counted as “incapacitated”. In addition, total fly number was recorded. Percentage Avoidance was calculated by determining the percentage of flies that avoid the noxious temperature compared to the total number of flies in the chamber. All tests were performed under low red light. Of note, this assay is an absolute measurement where flies that avoid the bottom heated surface were considered capable of noxious thermal avoidance, independent of how far they move away from the 46°C surface, though the vast majority of flies avoiding the hot surface were found on the top of the chamber. For experiments involving *UAS-Shibire^ts1^*, flies were transferred to the experimental chambers followed by a temperature shift to 30°C for 60 minutes. Larval pain assays were performed as described [7]. For assessing noxious temperature-induced paralysis, wild type flies were placed in 5 ml polystyrene round bottom tubes (BD Falcon, Germany) and exposed to temperatures ranging from 37–46°C with 1° increments) or only 46°C (for control vs *TrpA1* flies) and the temperature at which 100% of flies were paralyzed was recorded. General coordination was assessed by tapping the test chamber on the bench and observing activity as flies move away from the site of impact [35]. This response was quantified using a geotactic repulsion assay where flies were knocked to the bottom of a 15 ml polystyrene tube and the geotactic response (number of flies climbing up the tube / total number of flies) was recorded.

### Confocal microscopy

*Drosophila* brains were dissected in PBS, fixed in 4% paraformaldehyde in PBS for 30 min at room temperature (RT), washed three times for 10 min in PBS containing 0.3%Triton X-100, blocked for 1 hr at RT in PBT containing 5% normal goat serum, and incubated with primary anti-GFP (Sigma) and NC82 (Iowa Hybridoma Bank) counterstain antibodies in blocking solution overnight at 4 C. Samples were washed three times for 10 min in PBT at RT, and secondary antibodies were applied in blocking solution for 2 hr at RT. After washing three times for 10 min in PBS, samples were mounted in Vectashield (Vector Labs). Confocal images were captured on a Zeiss LSM510 Meta, Axiovert 200 M, and processed with LSM510 Image Examiner.

### Hypergeometric enrichment test

A hypergeometric test, similar to the test used for GO enrichment analysis, was used to identify over-represented gene lists (C2 from Msigdb, BROAD Institute) and pathways (KEGG) amongst the pain hits. The hypergeometric test considers only the percentage representation of genes corresponding to a biological pathway in the pre-computed heart function gene list. This analysis was performed on the gene list identified as adult pain hits (Z-score>1.65) in *Drosophila* and their corresponding mouse or human orthologs.

### Statistics

For analysis of adult *Drosophila* avoidance responses a Student's t test was performed. For analysis of larval pain behavior we have performed the Kruskal-wallis non-parametric test for median comparison followed by the Dunn's post-hoc test. For presentation of screening data, a Z-score was generated from (mean control avoidance − mean test avoidance)/standard deviation control) and P values were generated from total Z-score distributions. Unless otherwise indicated, data are represented as mean values ± SEM.

## Supporting Information

Table S1 **Ca^2+^ signalling involved in thermal nociception responses.** Ca^2+^ signaling components (*elav-Gal4>UAS-IR* fly lines) that exhibit defects in thermal nociception are listed from the strongest to weakest phenotype for noxious heat avoidance. An avoidance Z-score +/− SEM and repetitions are included. Lethality was scored for each cross (0 = lethal, 0.5 = semi-lethal, 1 = viable). A mean score of ≤0.6666 was considered lethal. A qualitative coordination score (0 = uncoordinated, 1 = coordinated) +/− SEM and number of repetitions are indicated for the re-screened lines. The CG numbers according to flybase annotation version 4.3 (FB4.3) and flybase annotation 5.7 (FB5.7) are included, as is the S19 score for RNAi off targeting effects (OTEs) and number of can repeats. A *p-value* is also included. *Drosophila* gene symbols and predicted human and mouse orthologs are shown.
(XLSX)
